# The Importance of Describing Patient Populations

**DOI:** 10.1177/15269248241237823

**Published:** 2024-03-06

**Authors:** Mary K. Roberts, Jonathan Daw

**Affiliations:** 1 Department of Sociology and Criminology, 8082 The Pennsylvania State University, University Park, PA, USA; 2 8082 The Pennsylvania State University, University Park, PA, USA

**Keywords:** populations, sample characteristics, descriptive, demography, statistical inference

Many clinical research articles struggle to articulate the relevant distinctions between their sample, their available population, and their target population. Perhaps you have written some of them. These distinctions are critically important to establish the external validity of a given dataset and analysis, particularly when one's sample is limited to patients at one or a handful of medical institutions and restricted to patients willing to participate. Here, we provide some succinct background on this topic and some specific advice.

The primary meaning of population is people living in a particular place and time (eg, everyone living in the United States in 2023).^1^ Researchers are often unable to obtain actual parameters (numerical characteristics) of a population, and thus must rely on sample statistics to characterize the total population of interest (subject to sampling error). The sample researchers can examine is a subset of people selected from the population, and when selecting the sample researchers often strive to have samples with similar demographic compositions and other relevant characteristics as the population of interest. In survey research, this is often achieved through survey weighting, counting underrepresented respondents in the sample more highly than other respondents so that the weighted sample characteristics match the population characteristics (measured, for example, in the Census).

As a clinical researcher, however, you have 2 advantages over other types of sample-based research. You typically have some idea of the distribution of key characteristics of the accessible patient population associated with the medical institution(s) where you are collecting the sample. This advantage should not be taken for granted, as researchers in other fields frequently struggle to directly characterize the population of interest when no registry or administrative dataset exists to describe them (known as hidden populations). Of course, for many research purposes you could use these administrative and health record datasets as a sampling frame for your primary data collection.

**Figure 1.**
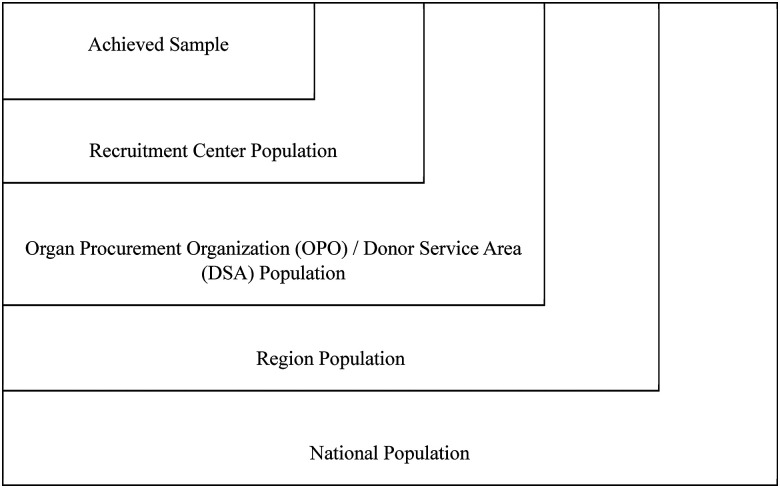
Visual representation of achieved sample and nested administrative structures.

Second, in the case of transplantation research, the patient population is nested in a series of administrative structures that yield a series of natural comparisons to help researchers understand the relationships between the achieved sample and the target population (see **Figure 1**). Consider your sample at the end of your data collection process. Hopefully, you have gathered some information about them that can be compared to the same characteristics among patients who did not participate in your study in the same center(s). This is comparing your achieved sample to your accessible population. Furthermore, your center(s) of recruitment are nested within organ procurement organization donor service areas, which are nested within UNOS regions, which together cover the full United States. Because the distribution of so many characteristics differ geographically, it is important to conduct these comparisons in a manner that respects these nested data structures to ensure you reach accurate conclusions.

**Table 1** provides age and race/ethnicity compositions of unique candidates for kidney transplantation added to the waitlist from 2018 to 2022 for the nation, region 2 (Delaware, D.C., Maryland, New Jersey, Pennsylvania, Virginia, and West Virginia), and 2 centers within the region (Hospital of the University of Pennsylvania and Charleston Area Medical Center). These data are based on OTPN data as of November 22, 2023 (http://optn.transplant.hrsa.gov). If a study were to draw samples representative of these centers, the sample characteristics of each center would be different from each other, and different from the region and nation they are nested within. For example, Charleston Area Medical Center demographics underrepresents the non-Hispanic Black (10.50%) compared to the Hospital of the University of Pennsylvania (37.40%), the region (37.14%), and nation (28.88%). Both centers underrepresent Hispanics (6.54% and 0.42%), compared to the region (9.78%) and the nation (19.76%). Charleston overrepresents the 65 +  population (30.67%) compared to the other center, region, and nation (16.70%, 23.65%, and 20.85%, respectively). Moreover, neither center represents any of the pediatric transplant candidates in the region or nation.

**Table 1.** Age and Race/Ethnicity Composition of Kidney Transplant Candidate Waitlist Additions From 2018 to 2022.

	National		Region 2		Hospital of the University of Pennsylvania		Charleston Area Medical Center	
	N	%	N	%	N	%	N	%
**Age**								
<1 year	38	0.02	4	0.02	0	0.00	0	0.00
1-5 years	1089	0.55	125	0.52	0	0.00	0	0.00
6-10 years	848	0.43	79	0.33	0	0.00	0	0.00
11-17 years	3306	1.67	368	1.53	0	0.00	0	0.00
18-34 years	21 395	10.79	2194	9.10	305	12.70	33	6.93
35-49 years	49 493	24.95	5506	22.83	617	25.70	102	21.43
50-64 years	80 996	40.83	10 155	42.11	1079	44.94	195	40.97
65+	41 362	20.85	5704	23.65	401	16.70	146	30.67
**Race/Ethnicity**								
White-NH	82 742	41.71	10 891	45.16	1141	47.52	416	87.39
Black-NH	57 289	28.88	8961	37.16	898	37.40	50	10.50
Hispanic/Latino	39 191	19.76	2359	9.78	157	6.54	2	0.42
Asian-NH	15 170	7.65	1776	7.36	198	8.25	6	1.26
AINA-NH	1639	0.83	40	0.17	2	0.08	0	0.00
Pacific Islander-NH	1029	0.52	32	0.13	4	0.17	2	0.42
Multiracial-NH	1780	0.90	100	0.41	1	0.04	0	0.00

As this table illustrates, centers may differ substantially from one another and UNOS regions differ considerably from the nation as a whole. Thus, it is necessary to compare your achieved sample to your accessible population (center[s] of recruitment), your accessible population to its UNOS region, and UNOS region to the nation to assess how successful your study was at collecting a representative sample of your available and target populations. By comparing the sample characteristics to those of the different populations it is nested within allows the researcher to show whether (or not) their findings are generalizable beyond the achieved sample.
